# Supplementary material for: A high-resolution mRNA expression time course of embryonic development in zebrafish
Source: eLife. 2017 Nov 16;6:e30860. doi: 10.7554/eLife.30860 (PMC5690287; doi:10.7554/eLife.30860)
Supplement: Supplementary file 6. [file elife-30860-supp6.zip › biolayout-clusters-files/Cluster220-genes.html]

Cluster220


# Cluster220: Genes

| Ensembl ID | Gene Name | Chr | Start | End | Biotype |
| --- | --- | --- | --- | --- | --- |
| ENSDARG00000079004 | ENSDARG00000079004 | 12 | 3199927 | 3217223 | protein\_coding |
| ENSDARG00000079182 | NPAS3 | 17 | 9691075 | 9734037 | protein\_coding |
| ENSDARG00000020926 | creb3l3l | 8 | 31365887 | 31375388 | protein\_coding |
| ENSDARG00000099563 | inpp5ka | 15 | 33985617 | 34007050 | protein\_coding |
| ENSDARG00000059960 | plch2a | 8 | 47497108 | 47633527 | protein\_coding |
| ENSDARG00000040123 | zfpm2a | 16 | 43871448 | 44179878 | protein\_coding |
